# Expert opinion on screening, diagnosis and management of diabetic peripheral neuropathy: a multidisciplinary approach

**DOI:** 10.3389/fendo.2024.1380929

**Published:** 2024-06-17

**Authors:** Aysegul Atmaca, Aysegul Ketenci, Ibrahim Sahin, Ihsan Sukru Sengun, Ramazan Ilyas Oner, Hacer Erdem Tilki, Mine Adas, Hatice Soyleli, Tevfik Demir

**Affiliations:** ^1^ Department of Endocrinology and Metabolism, Ondokuz Mayis University Faculty of Medicine, Samsun, Türkiye; ^2^ Department of Physical Medicine and Rehabilitation, Koc University Faculty of Medicine, Istanbul, Türkiye; ^3^ Department of Endocrinology and Metabolism, Inonu University Faculty of Medicine, Malatya, Türkiye; ^4^ Department of Neurology, Dokuz Eylul University Faculty of Medicine, Izmir, Türkiye; ^5^ Department of Internal Medicine, Adiyaman University Faculty of Medicine, Adiyaman, Türkiye; ^6^ Department of Neurology, Ondokuz Mayis University Faculty of Medicine, Samsun, Türkiye; ^7^ Department of Endocrinology, Prof. Dr. Cemil Tascioglu City Hospital, Istanbul, Türkiye; ^8^ Department of Medical Affairs, Abdi Ibrahim Pharmaceuticals, Istanbul, Türkiye; ^9^ Department of Endocrinology and Metabolism, Dokuz Eylul University Faculty of Medicine, Izmir, Türkiye

**Keywords:** diabetic peripheral neuropathy, expert panel, multidisciplinary approach, pathogenesis, awareness, screening, clinical diagnosis, pathogenetically-oriented pharmacotherapy

## Abstract

The proposed expert opinion aimed to address the current knowledge on conceptual, clinical, and therapeutic aspects of diabetic peripheral neuropathy (DPN) and to provide a guidance document to assist clinicians for the best practice in DPN care. The participating experts consider the suspicion of the disease by clinicians as a key factor in early recognition and diagnosis, emphasizing an improved awareness of the disease by the first-admission or referring physicians. The proposed “screening and diagnostic” algorithm involves the consideration of DPN in a patient with prediabetes or diabetes who presents with neuropathic symptoms and/or signs of neuropathy in the presence of DPN risk factors, with careful consideration of laboratory testing to rule out other causes of distal symmetric peripheral neuropathy and referral for a detailed neurological work-up for a confirmative test of either small or large nerve fiber dysfunction in atypical cases. Although, the first-line interventions for DPN are currently represented by optimized glycemic control (mainly for type 1 diabetes) and multifactorial intervention (mainly for type 2 diabetes), there is a need for individualized pathogenesis-directed treatment approaches for DPN. Alpha-lipoic acid (ALA) seems to be an important first-line pathogenesis-directed agent, given that it is a direct and indirect antioxidant that works with a strategy targeted directly against reactive oxygen species and indirectly in favor of endogenous antioxidant capacity for improving DPN conditions. There is still a gap in existing research in the field, necessitating well-designed, robust, multicenter clinical trials with sensitive endpoints and standardized protocols to facilitate the diagnosis of DPN via a simple and effective algorithm and to track progression of disease and treatment response. Identification of biomarkers/predictors that would allow an individualized approach from a potentially disease-modifying perspective may provide opportunities for novel treatments that would be efficacious in early stages of DPN, and may modify the natural course of the disease. This expert opinion document is expected to increase awareness among physicians about conceptual, clinical, and therapeutic aspects of DPN and to assist them in timely recognition of DPN and translating this information into their clinical practice for best practice in the management of patients with DPN.

## 1 Introduction

The International Diabetes Federation 2021 report considers diabetes a fast-growing global pandemic of the 21^st^ century, estimating an increase in the prevalence from 10.5% (536.6 million people) in 2021 to 12.2% (783.2 million people) in 2045 (1).

The continuing rise in diabetes and prediabetes burden worldwide is expected to manifest itself also in the form of increasing prevalence of chronic diabetes-related complications, such as diabetic nephropathy, within the next decades (2–5). Diabetic neuropathy is one of the most common and troublesome complications of both type 1 diabetes (T1D) and type 2 diabetes (T2D), while patients with prediabetes may also develop diabetic neuropathy, which ultimately advances upon transition to frank T2D (2–5).

Neuropathy in diabetes constitutes a heterogeneous group of disorders that differ with respect to clinical presentations and the pathophysiological background, which can be categorized as “diffuse or symmetrical” neuropathies (distal symmetrical polyneuropathy, autonomic and acute sensory neuropathy) and “focal or multifocal” neuropathies (radiculoplexus neuropathy, entrapment syndromes, cranial palsies and other mononeuropathies) (2–4). Still, patients with diabetes may also develop non-diabetic neuropathies (pressure palsies, acute treatment-induced painful small fiber neuropathies and chronic inflammatory demyelinating polyneuropathy) (2, 6).

By far, the most common diabetic neuropathy is distal symmetric polyneuropathy (also known as typical diabetic neuropathy), which we will primarily focus on and refer to as diabetic peripheral neuropathy (DPN) in this review (3, 7). Accounting for approximately 75% of all diabetic neuropathies, DPN affects up to 50% of T1D and T2D patients and at least 10% of prediabetic patients, while there is heterogeneity and wide variation in prevalence estimates depending on the applied diagnostic methodology (4, 8–12). DPN refers to damage to peripheral nerves including characteristic glove and stocking-like presentation of distal sensory or motor function loss with or without neuropathic pain (3, 4, 7, 9, 13, 14).

Untreated DPN has substantial effects on patient morbidity and quality of life (i.e., loss of limb sensation, falls, and increased risk of foot ulcers and lower limb amputations), while diabetic patients with DPN are also at higher risk of all-cause and cardiovascular mortality than those without DPN (3, 5, 7, 15–17).

Accordingly, early recognition and preventive measures are essential in DPN practice (3, 5, 7).

However, in contrast to other major diabetes complications (i.e., retinopathy and nephropathy), no single gold standard diagnostic test exists for DPN, along with diagnostic challenges particularly for diagnosing DPN early in the disease course (3).

Besides, the management of DPN primarily relies on improved glycemic control, which is more effective in T1D than in T2D, lifestyle and multifactorial risk interventions (mainly in T2D), while the symptomatic management in painful DN enables sufficient pain relief in less than one-third of patients (3, 4, 18). Hence, there is a need for specific disease-modifying treatments and standardized validated tools to identify risk groups in terms of sub-clinical neuropathy, disease mechanisms and treatment response that would enable the implementation of personalized treatment/mechanism-based approaches (4, 18).

Therefore, the proposed expert opinion aims to address the current knowledge on conceptual, clinical, and therapeutic aspects of DPN and to provide a practical guidance document to assist clinicians in the best practice in recognition, diagnosis, and management of the disease.

## 2 Methods

The present expert panel of four different specialties involved in the management of DPN (endocrinology, neurology, internal medicine and physical medicine and rehabilitation) with at least 15 years of experience in the management of DPN, convened a meeting to develop a consensus opinion on the conceptual, clinical and therapeutic aspects of DPN. The panel critically analyzed recommendations from existing guidelines and data from systematic reviews, meta-analyses and literature reviews of articles published on DPN and agreed on a series of statements supported by scientific evidence and expert clinical opinion to assist clinicians on best practices in recognition, diagnosis, and management of DPN. The proposed expert opinion planned to provide a practical and implementable guidance document addressing DPN in terms of:

a) definition, pathophysiology and risk factors,
b) clinical manifestations and diagnosis (current challenges, a proposed screening and diagnostic algorithm),
c) screening for early diagnosis and symptom progression, and
d) management of disease in terms of the optimal diabetes treatment (intensive glycemic control, lifestyle modification and multifactorial risk intervention), the pathogenetically oriented pharmacotherapy (with special emphasis on alpha lipoic acid), and the symptomatic pain relief (in painful DPN).

## 3 Definition, pathophysiology, risk factors of DPN

### 3.1 Definition

DPN is considered a symmetrical, length-dependent sensorimotor polyneuropathy attributable to metabolic and microvascular alterations due to chronic hyperglycemia exposure (diabetes) and cardiovascular risk covariates (13, 19). A simpler definition for clinical practice can be ‘the presence of symptoms and/or signs of neuropathy that develops in the context of prediabetes or diabetes after the exclusion of other causes of peripheral neuropathy (20–22).

DPN progresses gradually with increasing duration of diabetes and in relation to glycemic control in both T1D and T2D (23, 24). Notably, while the prevalence of DPN is considered to be rather low within the first five years of disease onset in T1D, slowing of nerve conduction velocity may be one of the earliest neuropathic abnormalities in T2D, often present at diagnosis (23, 24). Hence, DPN should be suspected in all patients with prediabetes and T2D and those who have had T1D for more than five years (24, 25).

### 3.2 Pathophysiology

Although the exact pathophysiology of DPN remains unknown, the role of various signaling cascades has been proposed such as advanced glycation end-product (AGE) pathway, polyol pathway, protein kinase C (PKC) pathway, hexosamine pathway and poly (ADP ribose) polymerase (PARP) pathway (9, 26–29). Endothelial health is compromised by multiple factors such as glycosylation, hyperlipidemias, hyperhomocysteinemia, hypertension, excessive platelet activity, reduced nitric oxide and excessive generation of reactive oxygen species (ROS) (30, 31).

Nonetheless, chronic hyperglycemia is the main underlying cause of DPN, which leads to damage at the vascular level and the free passage of glucose to neurons and Schwann cells, resulting in an imbalance between nerve damage and nerve fiber repair (20, 32). All the proposed pathways in pathogenesis are activated with hyperglycemia, and they can directly or indirectly impair the redox capacity of the cell and increase the production of ROS (9, 26–29). With the accumulation of ROS (oxygen free radicals such as superoxide anion radical and hydroxyl radical), the endogenous antioxidant defense system (i.e., superoxide dismutase, catalase, glutathione ascorbate) fails to counteract ROS generation, resulting in an increase in oxidative stress (28, 33). The increased oxidative stress leads to impaired neural function, gradually heading to apoptosis in neurons and supporting glial cells (i.e., Schwann cells and satellite glial cells) of the peripheral nervous system (9, 26–29).

The causes of DPN are thought to be a multi-factorial metabolic process that increasingly deteriorates tissues. Hyperglycemia, increased sorbitol and protein kinase C, elevated homocysteine, reduced nitric oxide and excessive Reactive Oxygen Species (ROS) damage endothelial tissue and produce a rheological change that increases vascular resistance and reduces blood flow to the nerve

### 3.3 Risk factors

Diabetes is the strongest risk factor for DPN, along with certain disease characteristics such as diabetes duration and poor glycemic control in terms of fasting blood glucose (FBG), glycated hemoglobin (HbA1c; >7%), postprandial hyperglycemia and the glycemic variability (4, 7, 25, 34–39). Poor glycemic control is the most critical and principal modifiable risk factor for DPN (4, 5, 7, 25). Presence of symptoms and/or signs of distal symmetric polyneuropathy have also been reported in prediabetes although prevalence is variable among studies (40–42). A growing body of evidence supports an association between prediabetes and early small-fiber symptoms and the likelihood of neuropathy to be already developed in the prediabetic stage (7, 12). Given the stronger association of neuropathy with impaired glucose tolerance (IGT) rather than the impaired fasting glucose (IFG), post-load hyperglycemia seems to be the key mechanism for inducing increased oxidative stress, endothelial dysfunction, and activation of both PKC and the polyol pathway, leading to impaired neuronal metabolism and DNA damage in prediabetic stage (37, 43).

Other risk factors increasing the prevalence of DPN include the non-modifiable risk factors such as older age (>50 years), height (directly related to the length of the axon), female gender (particularly for painful DPN), comorbid diabetic retinopathy/nephropathy and positive HLA-DR3/4 genotype, and the modifiable risk factors including the hypertension, dyslipidemia, smoking, alcohol use, obesity, and vitamin D deficiency, vitamin B12 deficiency and low C-peptide levels (4, 7, 25, 32–37, 44–49) (**Table 1**). The recognition of risk factors is of critical importance in the timely identification of subclinical/early DPN for effective disease control and prevention of hazards (i.e., ulcer, gangrene, and amputation) and social burden (2, 32, 50).

**Table 1:** Risk factors of DPN (4, 7, 12, 25, 32–37, 43–47).

Unmodifiable risk factors
Advanced age (>50 years)
Duration of diabetes
Height
Positive HLA-DR3/4 genotype
Presence of other microvascular complication (i.e., diabetic retinopathy and nephropathy)
Modifiable risk factors
Poor glycemic control (HbA1c, fasting plasma glucose, postprandial hyperglycemia, glycemic variability)
Obesity (body mass index, weight, waist circumference, waist to hip ratio)
Dyslipidemia (hypertriglyceridemia, high LDL, low HDL)
Hypertension
Smoking
Heavy alcohol intake
Low C-peptide levels
Vitamin D and Vitamin B12 deficiency

HbA1c, glycated hemoglobin; HDL, High-density lipoprotein; LDL, low-density lipoprotein cholesterol

## 4 Clinical manifestations, screening and diagnosis of DPN

### 4.1 Clinical manifestations

DPN usually manifests as a length-dependent, distal-symmetrical, sensorimotor polyneuropathy (22, 51). Its onset is generally insidious, and the course is chronic and progressive without treatment (23). Symptoms at presentation can be either “positive”, including neuropathic pain (described as deep aching, burning, and sharp stabbing sensations), paresthesia and hyperesthesia, or “negative”, including loss of sensations (hypoesthesia), including different sensory modalities relating to small fiber (temperature, pain) and large fiber (touch, pressure, vibration, position) functions and ataxic gait (6, 20, 22, 52, 53).

Alternatively, some patients may be completely asymptomatic, and signs may be only revealed by a detailed neurological examination (6, 22, 23), while some others may present with rare, atypical diabetic neuropathies with distinct features and underlying mechanisms, as well as with neuropathies attributed to causes other than diabetes (6, 54).

In accordance with the higher vulnerability of lower-limb long axons to injury, DPN usually develops first in the feet and then progresses proximally involving the upper limbs (dying-back type of axonal degeneration), and patients typically present with a “stocking-glove” like distribution of neuronal dysfunction (20, 22, 51).

Overall, up to 50% of affected subjects do not report symptoms, and up to one fourth of patients develop painful DPN which is particularly associated with physical and psychosocial impairment, disability, and reduced health-related quality of life (20–22, 24, 25, 55).

Most patients show a “mixed” neuropathy with both large and small nerve fiber damage and all patients with DPN are considered to be at increased risk of neuropathic complications such as foot ulceration and Charcot’s neuroarthropathy as well as the falls and fractures (20, 23–25).

### 4.2 Underdiagnosis – insufficient awareness among physicians

Despite its major impact on morbidity and mortality, DPN is frequently underdiagnosed and underestimated in clinical practice (22, 23, 56, 57). The factors considered responsible for the challenges in recognition and timely diagnosis of DPN include:

a) the insidious course of disease manifesting with non-specific symptoms and signs mimicking many other diseases,
b) a lack of consensus on optimal screening and diagnostic procedures,
c) the absence of a well-established diagnostic scrutiny, and
d) the poor acceptance of guidelines and insufficient awareness of the disease among physicians (22, 23, 25, 30, 56–58).

DPN is a diagnosis by exclusion, and its recognition is mainly based on clinical suspicion and the effort put into finding it (22, 23). However, there is insufficient physician awareness regarding the recognition of DPN, even when the neuropathy is symptomatic (22, 23, 30, 57, 58). The population-based studies reported that painful DPN and painless DPN were previously undiagnosed in almost 60% and 80% of T2D patients, respectively (59–61).

The current practice of DPN care is also considered inadequate in terms of patient journey which includes multiple visits to different clinicians having no specialist training to assess the level of risk, to provide advice, or to appropriately refer the patients for further investigation and appropriate interventions (62, 63).

Accordingly, the participating experts consider the suspicion of the disease by clinicians as the key factor, emphasizing the awareness of the disease during the patient journey by the first-admission or referring physicians. In this regard, improved awareness of DPN among internal medicine, family medicine and physical medicine and rehabilitation specialists besides the neurology and endocrinology specialists as the most consulted specialists, seems to be the key factor in consideration of DPN in differential diagnosis of patients presenting with suspected neuropathic symptoms. Increase in familiarity of non-specialist physicians with recognition of DPN seems also important to reduce the unnecessary referrals among several different specialists before a correct diagnosis is reached.

### 4.3 Rationale for screening

DPN is considered to appear in approximately half of all diabetic individuals at some stage in their lives, emphasizing the utility of screening in early diagnosis of DPN (9, 64). Data from the MONICA/KORA Augsburg Surveys from Germany revealed the varying prevalence of DPN and painful DPN across the spectrum of diabetes, pre-diabetic states and normoglycemia, including known diabetes (28% and 13%, respectively), IGT (13% and 9%), IFG (11% and 4%) and normal glucose tolerance (NGT; 7% and 1%) (40, 65).

In a multicenter study with 1,113 diabetic patients from Turkey, the prevalence of DPN was found to be 40.4% based on the clinical examination alone and increased to 62.2% by combining nerve conduction studies with clinical examination, while the neuropathic pain prevalence was 14.0% (66). In another study from Turkey in 100 newly diagnosed prediabetic individuals who were screened for microvascular and macrovascular diabetic complications, microvascular complications were found in 12% of the participants (neuropathy: 4%, nephropathy: 8%) and 19% had macrovascular complications (67).

Accordingly, given the accumulating evidence on the increasing risk of DPN in patients with prediabetes and with the duration of the disease in those with known diabetes (40, 65, 68), early screening for DPN in the setting of prediabetes and diabetes is important to prevent and delay the occurrence of DPN (20, 22, 34). Screening for early detection and subsequent follow-up of progression is also important given that DPN is already well-established by the time its symptoms and/or clinical signs develop, impeding the benefits of intensified multifactorial intervention at an early stage of disease trajectory (3, 63, 69, 70).

The newly updated “American Diabetes Association (ADA) Standard of Care in Diabetes 2024” for Neuropathy Screening Guidelines states that patients with T2D at the time of diagnosis and those with T1D five years after diagnosis should be evaluated for DPN by obtaining a careful medical history and physical examination (sensory assessment and foot examination), and subsequently they should be evaluated every year (71).

In addition, given the likely association between different microvascular complications, as well as between micro and macrovascular complications of diabetes, when a patient is diagnosed to have one diabetes complication, it is important to screen for others (72, 73).

### 4.4 A proposed screening and diagnostic algorithm

Clinical history and examination are the mainstays of clinical diagnosis, and when medical history and basic neurological examination (simple semi-quantitative bedside instruments and inspection of feet) reveals the corresponding neuropathic symptoms and/or signs in the absence of other potential causes of distal symmetric peripheral neuropathy, this supports the diagnosis of DPN (4, 6, 7, 23, 74).

Accordingly, in most cases, the diagnosis of DPN is made by ruling out other potential causes of distal symmetric peripheral neuropathy, such as alcohol use, nutritional deficiencies (i.e., vitamins B6, B12, and E, thiamine, folate, copper, phosphate), hypothyroidism/hyperthyroidism, autoimmune diseases (i.e., systemic lupus erythematosus, rheumatoid arthritis), malignancy (i.e., multiple myeloma), infections (HIV/AIDS, Lyme disease), chemotherapy and toxin exposure (4, 11, 20, 49, 72). This necessitates a careful medical history and a screening laboratory test for complete blood count, comprehensive metabolic profile, fasting blood glucose, thyroid-stimulating hormone, and vitamin B12 levels, as well as the serum protein electrophoresis with immunofixation to exclude other causes of peripheral neuropathy (6, 20, 22, 25, 56, 63, 75, 76).

**Figure 1:**
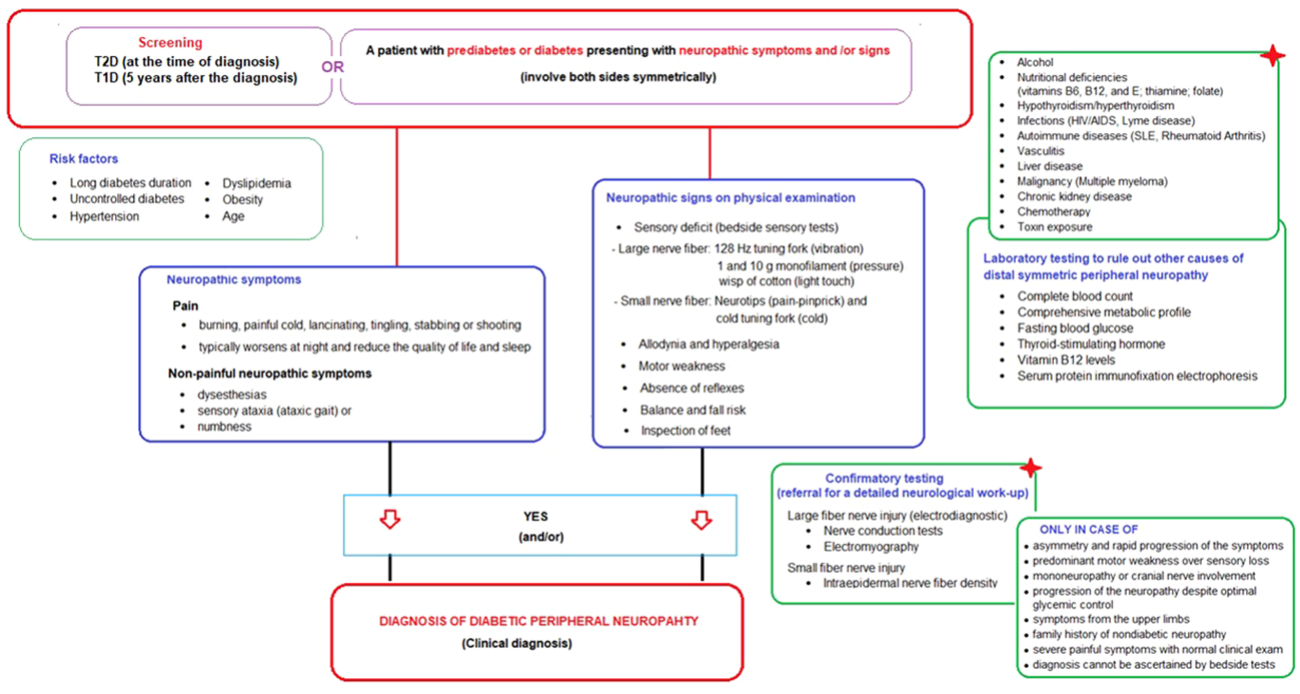
Screening and diagnostic algorithm for diabetic peripheral neuropathy in prediabetic and diabetic patients presenting with neuropathic symptoms and/or signs.

*Neuropathic symptoms* include the pain (characteristically described as burning, painful cold, lancinating, tingling, stabbing or shooting) and the non-painful neuropathic symptoms such as paresthesias (tingling, prickling or ant-like sensations), dysesthesias (unpleasant abnormal sensation whether spontaneous or evoked), sensory ataxia (ataxic gait) or numbness (often described as “wrapped in wool” or like “walking on thick socks”) (20, 22) (**Table 2**; **Figure 1**).

**Table 2:** Neuropathic symptoms/signs: Medical history and neurological examination (20, 22, 23).

Neuropathic symptoms - medical history			
Pain: burning, painful cold, lancinating, tingling, stabbing or shooting (electric shock–like)			
Non-painful symptoms • Paresthesias (tingling, prickling or ant-like sensations) • Dysesthesias (unpleasant abnormal sensation whether spontaneous or evoked) • Sensory ataxia (ataxic gait) • Numbness (often described as “wrapped in wool” or like “walking on thick socks”)			
Neuropathic signs - basic neurological examination			
1) Sensory deficit via bedside sensory tests^*^			
*Sensory modality*	*Nerve Fiber*	*Instrument*	*Sensory receptors*
Vibration	Aβ (large)	128 Hz Tuning fork	Ruffini corpuscle mechanoreceptors
Pain (pinprick)	C (small)	Neurotips	Nociceptors for pain and warmth
Pressure	Aβ, Aα (large)	1 and 10 g Monofilament	Pacinian corpuscle
Light touch	Aβ, Aα (large)	Wisp of cotton	Meissner’s corpuscle
Cold	Aδ (small)	Cold tuning fork	Cold thermoreceptors
**2) Other neuropathic signs**			
Allodynia, hyperalgesia, motor weakness, absence of ankle and patellar deep tendon reflexes, balance and fall risk			
**3) Inspection of feet**			
Deformities, ulcers, fungal infection, muscle wasting, hair distribution or loss, presence or absence of pulses			

^*^Sensory receptors are innervated by different types of nerve fibers. The proprioceptors are innervated by type Aα (type Ia, Ib) and type Aβ (type II) sensory fibers, the mechanoreceptors by type Aβ (type II) and type Aδ (type III) sensory fibers, and the nociceptors and thermoreceptors type Aδ (type III) and C (type IV) sensory fibers. Type Aα fibers include the type Ia and type Ib sensory fibers from muscle spindle endings and the Golgi tendon, respectively. Type Aβ fibres are the type II afferent fibers from stretch receptors, and those from the skin are mostly dedicated to touch. Type Aδ fibers are the afferent fibers of nociceptors which carry cold, pressure, and acute pain signals and because they are thin (2–5 μm in diameter) and myelinated, they send impulses faster than unmyelinated C fibers, but more slowly than other, more thickly myelinated group A nerve fibers.

*Neuropathic signs* detected on basic neurological examination including bedside sensory tests and foot inspection include (a) sensory deficit in large nerve fiber (via 128 Hz tuning fork for vibration sensation, 1 and 10 g monofilament for pressure sensation and wisp of cotton for light touch sensation), (b) sensory deficit in small nerve fiber (via neurotips for pain-pinprick sensation) and cold tuning fork for cold sensation), (c) allodynia (pain triggered by normally non-painful stimuli such as the contact of socks, shoes, or bedclothes), (d) hyperalgesia (exaggerated response to painful stimuli), (e) motor weakness (extension of the big toe, ankle dorsiflexion, and walking on heels), (f) absence of reflexes (ankle and patellar deep tendon reflexes), (g) balance and fall risk (Romberg test, normal gait, and tandem gait), and (h) inspection of feet (for deformities, ulcers, fungal infection, muscle wasting, hair distribution or loss, and the presence or absence of pulses) (20, 22, 23) (**Table 2**; **Figure 1**).

Small and large nerve fiber damage most frequently coexist in DPN, and thus testing small and large nerve fiber function with appropriate semi-quantitative bedside tests is equally important (22, 51). In the painful DPN, intensity of pain is evaluated via numeric rating scale [NRS] or a visual analogue scale [VAS]), while the pain typically worsens at night and may interfere with daily activities and reduce the quality of life and sleep and characteristics (20, 22, 23).

The ADA definition does not require an abnormal electrodiagnostic test for clinical neuropathy diagnosis, as also supported by studies indicating that electrodiagnostic tests rarely change the etiology and/or management of patients meeting a clinical DPN definition (3, 77, 78). However, when evaluating a person with diabetes and signs and/or symptoms of neuropathy, it is important to remember that DPN is not the only possible cause of these signs and/or symptoms and that rare diabetic neuropathies or even neuropathies of other etiologies may be present (6, 24). Hence, the confirmatory testing (detailed neurological work-up) is not usually needed for clinical diagnosis but is used in clinical research and is helpful in the diagnosis of patients with atypical presentations (6, 7). These tests include the large fiber nerve injury tests (electrodiagnostic tests: nerve conduction tests and electromyography) and the small fiber nerve injury (intraepidermal nerve fiber density) (6, 7, 74) (**Figure 1**).

The atypical presentations that should alert the physician to consider non-diabetic causes of neuropathy via referral for a detailed neurological work-up (confirmatory tests) include: (1) asymmetry and rapid progression of the symptoms, (2) predominant motor weakness over sensory loss, (3) mononeuropathy or cranial nerve involvement, (4) progression of the neuropathy despite optimal glycemic control, (5) symptoms from the upper limbs, (6) family history of nondiabetic neuropathy, (7) severe painful symptoms in the feet whilst clinical examination is normal, 8) diagnosis of DPN cannot be ascertained by clinical examination with the semi-quantitative bedside tests (6–8, 20, 22, 74) (**Figure 1**).

Accordingly, the present expert panel suggests that DPN should be considered in a patient with prediabetes or diabetes who presents with neuropathic symptoms (involve both sides symmetrically) and/or signs of neuropathy in the presence of DPN risk factors (i.e., advancing age, obesity, hypertension, dyslipidemia, poor glycemic control), with careful consideration of laboratory testing to rule out other causes of distal symmetric peripheral neuropathy and referral for a detailed neurological work-up for a confirmative test of either small or large nerve fiber dysfunction only in atypical cases. The proposed “screening and diagnostic” algorithm is provided in **Figure 1**.

## 5 Screening tests and the challenges in clinical practice

Currently, there is no single gold standard test for objective assessment and early identification of DPN in routine clinical practice (28, 63), while there is considerable inter-physician variability in judgement and weighing of neuropathic symptoms/signs to draw a clinical diagnosis (3, 79).

Bedside sensory tests are operator-dependent tests that tend to diagnose DPN when it is already well-established, while their implementation is challenging by general practitioners or at busy diabetes clinics given the time constraints (28, 63, 80). Nevertheless, ADA recommends pinprick and temperature sensation tests for small fiber dysfunction; and lower extremity reflexes (particularly Achilles reflex) and vibration sensation with 128 Hz tuning fork for large fiber dysfunction (71). The expert panel also encourage the use of these tests (**Figure 1**).

Since the clinical-electrophysiological dissociation occurs regularly in the early stage of DPN, it is not uncommon to encounter diabetic patients with normal clinical but abnormal electrophysiological features (subclinical neuropathy) or those with abnormal clinical and normal electrophysiological features (early DPN; clinically defined DPN) (81).

Although the ADA definition does not require an abnormal electrodiagnostic test for clinical neuropathy diagnosis, nerve conduction studies are often needed to confirm the diagnosis and document the severity of DPN. Another essential clinical point is that the presence of DM in a patient with neuropathy does not prove that diabetes is the cause of polyneuropathy since it has been observed that neuropathy is due to causes other than diabetes in 2% of type 1 diabetics and 6% of type 2 diabetics (82). Moreover, nerve conduction studies could predict foot ulcers and even mortality (83).

DPN is the strongest risk factor for foot ulcers and extremity amputations, which lead to labor loss and have very high individual and social costs. Since DPN is a length-dependent process that occurs with sensory system dysfunction, examination of the dorsal sural and medial plantar nerves, whose responses are recorded more distally, their conduction studies are found to be much more sensitive than sural nerve conduction studies used in daily routine electrophysiological examination in detecting polyneuropathy (84, 85). Recently the abnormality of RR interval, another electrophysiological test, has been shown to provide early information about cardiac autonomic neuropathy, even without clinical abnormalities (86).

Nerve conduction tests and electromyography, provide higher sensitivity than clinical examinations in the evaluation of peripheral symmetrical polyneuropathies and are the least variable noninvasive measure of neuropathy and its progression (63, 81, 83, 87, 88). However, nerve conduction studies are labor-intensive, costly and impractical to implement in routine clinical care (22, 63). Moreover, DPN symptoms often occur with subclinical dysfunction of nerves. Skin biopsy is also used for diagnosing peripheral neuropathy on the basis of intraepidermal nerve fiber density (IENFD), while it is impractical for monitoring symptom progression or assessing treatment efficacy in clinical settings (28, 89).

The point-of-care devices (POCD), which are developed as rapid and non-invasive tests for diagnosis of subclinical neuropathy, include corneal confocal microscopy (CCM; for assessing small-fiber neuropathy and progression to large-fiber neuropathy), SUDOSCAN (for assessing sudomotor function), quantitative sensory testing (QST), NeuroQuick and NeuroPAD (28, 63, 90–93). Although the current evidence suggests the likelihood of PCODs to meet the criteria of an ideal screening test (safe, quick and sufficiently simple to provide objective measures), the cost of using these tests for screening purposes in all patients with diabetes and their convenience for busy diabetic clinics also need to be carefully appraised (63).

Over the years, a wide range of clinical scales, which often combine symptom assessments with bedside evaluations of clinical DPN signs, have been proposed as standardized objective and quantitative measures for screening and grading of severity of DPN (3, 52, 63). The most used ones are Michigan Neuropathy Screening Instrument (MNSI), Toronto Clinical Neuropathy Score (TCNS), United Kingdom Screening Test (UKST), Utah Early Neuropathy Score (UENS) and Neuropathy Impairment score for Lower Limbs (NIS-LL) (52, 63). However, their assessment remains subjective and heavily reliant on the examiners’ interpretations in addition to concerns regarding their validity, and they are considered particularly useful for epidemiological studies investigating the prevalence of DPN in larger populations (3, 52, 63, 94).

To improve clinical outcomes in DPN, there is an urgent need to diagnose DPN early before overt clinical signs are apparent and to assess disease progression accurately in order to effectively reduce morbidity and to reliably inform patients of their underlying risk of foot ulceration (63). Hence, rapid sensitive and specific tests that do not require specialty training but provide a reliable and sensitive cost-effective method to screen for DPN will ultimately be needed (3, 63, 79) (**Box 1**).

Box 1 **Key points - Screening tests in DPN (3, 22, 28, 52, 63, 79, 81–93).**

- There is no gold-standard test or specific simple markers for early detection of DPN in routine clinical practice.
- Bedside sensory tests are operator-dependent tests that tend to diagnose DPN when it is already well established and their implementation is challenging by general practitioners or at busy diabetic clinics given the time constraints.
- It is not uncommon to encounter diabetic patients with normal clinical but abnormal electrophysiological features (subclinical neuropathy) or with abnormal clinical and normal electrophysiological features (early DPN; clinically defined DPN).
- Nerve conduction tests and electromyography, are the least variable noninvasive measure of neuropathy and its progression, but they are labor-intensive, time-consuming, costly and impractical to implement in routine clinical care, and are unsuitable for evaluating the small-fiber neuropathy.
- Skin biopsy is used for diagnosing peripheral neuropathy on the basis of intraepidermal nerve fiber density (IENFD); however, it is impractical for monitoring symptom progression or assessing treatment efficacy in clinical settings.
- Pint-of-care devices (POCD), such as CCM (assessing the small-fiber neuropathy and progression to large-fiber neuropathy), SUDOSCAN (assessing the sudomotor function) and QST, are rapid and non-invasive tests for diagnosis of subclinical neuropathy. However, the cost of using these tests for screening purposes in all patients with diabetes and their convenience for busy diabetic clinics also need to be carefully appraised.
- The clinical scales are standardized objective and quantitative measures for screening and grading of severity of DPN but their results remain subjective and heavily reliant on the examiners’ interpretations in addition to concerns regarding their validity.
- There is an urgent need for rapid sensitive and specific tests that do not require specialty training to diagnose DPN early before overt clinical signs are apparent, to assess disease progression accurately in order to effectively reduce morbidity.

## 6 Management of diabetic peripheral neuropathy

DPN is best managed thorough by multidisciplinary support provided by an interprofessional team including endocrinology, internal medicine, neurology, physical therapy and rehabilitation, nephrology and ophthalmology specialists, a dedicated diabetic nurse, a dietician, an exercise specialist, a podiatrist, and a psychologist to ensure the provision of the available standard of care with minimal morbidity (8, 44, 63, 95, 96).

First-line interventions for DPN is currently represented by optimized glycemic control (mainly for T1D) and multifactorial intervention (mainly for T2D), along with lifestyle optimization and weight management (3, 97). There is a need for personalized treatment/mechanism-based approaches for DPN, which includes the targeted interventions based on the comorbid risk factors and underlying disease mechanisms specific to each patient, besides the optimal diabetes treatment (4, 18). Overall, the management of DPN is based three principles including (10, 22, 49, 97, 98):

(1) optimal diabetes treatment via intensive glycemic control, lifestyle modification and multifactorial risk intervention,
(2) pathogenetically oriented pharmacotherapy, and
(3) symptomatic pain relief

### 6.1 Optimal diabetes treatment: intensive glycemic control, lifestyle modification and multifactorial risk intervention

#### 6.1.1 Intensive glycemic control

Glycemic control early in the course of the disease is considered the most effective prophylactic treatment to delay the emergence of DPN, because hyperglycemia is the primary underlying pathophysiologic insult that contributes to DPN (9, 99).

However, while strict glycemic control slows DPN progression (78% relative risk reduction) in patients with T1D (100, 101), it has generally modest impact on DPN (5–9% relative risk reduction) in the setting of T2D, as reported by the large-scale studies (Steno 2, ADVANCE, ACCORD and VADT) and meta-analyses (102–107). This is considered in favor of the independent contribution of metabolic syndrome and its components (obesity, dyslipidemia, and hypertension), besides the hyperglycemia, to the onset and progression of DPN in T2D (5, 9, 25, 33, 45, 107, 108). However, since the DPN was not a primary outcome in the above-mentioned clinical trials, most of these studies relied on insensitive measures of neuropathy (9, 63, 106). Notably, the studies using more sensitive clinical endpoints (i.e., corneal confocal microscopy) revealed a benefit of strict glycemic control (HbA1c < 6.5%) also in T2D patients (63, 109, 110).

Moreover, the BARI 2D trial demonstrated a significantly lower cumulative incidence of DPN with the use of insulin-sensitizing agents (metformin, thiazolidinediones) rather than the insulin-providing (sulfonylurea and insulin) strategy (111). Hence, while glycemic control is the mainstay approach to preventing and managing diabetes-related complications, including DPN, the degree to which glycemic control can prevent or reverse DPN may differ depending on the choice of anti-diabetic agents used to achieve targets, type of diabetes and the duration of the disease as well as the glucose variability and glycemic excursions (4, 111, 112).

#### 6.1.2 Lifestyle modification and multifactorial risk intervention

Accumulating evidence indicates the multifactorial risk reduction strategies that involve adopting a healthy lifestyle incorporating a balanced diet (i.e., a low-fat, low-calorie diet, or a Mediterranean diet) and regular aerobic and weight-resistance physical activities as the best way to control the risk factors and prevent the development and progression of DPN (4, 7, 63, 113, 114).

RCTs in patients with DPN indicated the association of a 12-week exercise and lifestyle intervention with a significant reduction in DPN severity based on Modified Toronto Clinical Neuropathy Score [mTCNs] (115), and the association of a 2.5-hour, weekly supervised treadmill exercise and dietary intervention program with significant improvement in markers (intraepithelial nerve fiber density and regenerative capacity) of DPN (116).

Some authors also suggested that DPN in T2D is pathogenetically different from T1D and should be rather managed in the context of the metabolic syndrome (63, 97, 117). Nonetheless, several large intervention studies targeting multiple risk factors (UKPDS, Steno-2, ADDITION) failed to show a reduction in DPN despite clear benefits in renal and retinal complications (118–120). This, once again, emphasizes the importance of using sensitive, robust methods used to diagnose and quantify DPN (63, 97).

In this regard, further research is needed to re-examine the impact of multifactorial interventions upon DPN using more reliable, reproducible and sensitive measures of DPN (63). Given the growing need for personalized treatment/mechanism-based approaches that can consider individual differences in disease mechanisms, severity, and response to treatment (4, 18), early identifications of subjects with sub-clinical or early neuropathy using validated, yet novel non-invasive POCDs may allow investigation of the effect of a targeted intensified cardiometabolic risk factor control on prevention of clinical DPN or reversal of disease progression in larger studies (63).

In addition, while some studies indicate the association of angiotensin-converting enzyme (ACE) inhibitors with improvements in DPN based on clinical and nerve conduction parameters (121, 122), conflicting data have been reported on the impact of dyslipidemia treatment on DPN (45, 63, 97). Some studies found that treatment with either statin or fibrate was associated with a reduced risk of new-onset DPN and decreased the risk of neuropathy (43, 123, 124), while others indicated no association of statin therapy with a reduction of DPN risk (125) or even significantly higher prevalence of DPN among statin-users compared with non-users (126, 127). Nonetheless, the association of statins therapy with symptoms of DPN in diabetic patients remains a controversial subject, given that statin therapy is given for persons with a very prominent hazard of DPN, and there are also accessible substitutes of statins that are not associated with DPN (127).

### 6.2 Pathogenetically oriented pharmacotherapy

Upstream inhibition of key glycolytic enzymes by oxidative stress activates major pathways (polyol, hexosamine, PKC, PARP and AGE) implicated in the development of DPN (22, 128). Ideally, a targeted intervention is expected to address the underlying pathogenetic mechanisms and to alter the natural trajectory of the disease (97). Several compounds are available which target these major pathways implicated in the pathogenesis of DPN, such as aldose reductase inhibitors (sorbinil, tolrestat, ponalrestat, fidarestat, epalrestat, zenarestat) acting on polyol pathway, PKC inhibitors (ruboxiastaurin), benfotiamine acting on hexosamine pathway, agents acting on AGE pathway (minocycline, aminoguanidine), actovegin acting as PARP inhibitor and ROS inhibitors (alpha-lipoic acid, coenzyme Q10, nicotinamide, resveratrol, taurine) (52, 128).

Amongst them, alpha-lipoic acid (ALA) has a special place in the current DPN treatment paradigm, given that it is licensed as a drug for the treatment of DPN in many countries. Thus, its unique and wide-range antioxidant effects as a favorable agent aimed at the pathogenesis of DPN have been extensively investigated in countries where ALA is widely used, such as Germany, Eastern Europe and Far East (28, 31, 52, 99, 129).

#### 6.2.1 ALA as a disease-modifying pathogenesis-directed agent in DPN

**Figure 2:**
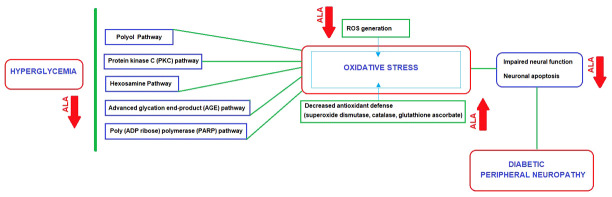
ALA as a disease-modifying agent: Direct and indirect antioxidant effects in relation to main pathways implicated in the DPN pathogenesis.

All of the pathways involved in DPN lead to a unique result of enhanced cellular oxidative stress caused by ROS generation and decreased antioxidant defense, which makes antioxidant response as an attractive drug target in DPN (130–132). ALA is considered a promising first-line disease-modifying antioxidant therapy for DPN in this regard, given its ability to prevent early development and progression of DPN by exerting direct (inhibition of ROS generation) and indirect (increase of endogenous antioxidant glutathione) antioxidant effects, which subsequently manifests as a notable clinical improvement of DPN (132–135) (**Figure 2**).Also, serum homocysteine level is considered a modifiable risk for DPN with significant contribution of hyperhomocysteinemia to endothelial tissue damage according to microvascular hypothesis (30, 136). ALA has been reported to ameliorate the hyperhomocysteinemia- induced endoplasmic reticulum stress and oxidation in human aortic endothelial cells (137). Hence, ALA may also be effective pathogenesis-directed agent in the setting of microvascular hypothesis in DPN, as a potentially homocysteine-lowering (30, 136).

In the ALADIN II (a-Lipoic Acid in Diabetic Neuropathy) trial, two years of initially intravenous and then oral administration of ALA was associated with improvement in motor and sensory nerve conduction velocity and amelioration of neuropathic symptoms (138).

In the SYDNEY 2 trial, oral 600 mg once daily treatment with ALA for 5 weeks improved neuropathic symptoms (stabbing pain, burning pain, paresthesia, and asleep numbness of the feet) and deficits in DPN as assessed by Neuropathy Symptoms and Change (NSC) and Neuropathy Impairment Score (NIS), providing the optimum risk-to-benefit ratio (139).

In a meta-analysis of three RCT trials in ALA-treated patients with symptomatic DPN, 600 mg ALA either orally or intravenously was shown to reduce significantly neuropathic symptoms (140). Another meta-analysis of four RCTs (ALADIN I, ALADIN III, SYDNEY, NATHAN I; in 1258 patients) demonstrated that 3 weeks of ALA therapy significantly improved the total symptom score (TSS; relative difference vs placebo: 24.1% with significant effect on pain, burning, and numbness), and the NIS-LL (relative difference versus placebo: 16.0% with significant effect on ankle reflexes, pin-prick and touch-pressure sensation) (141). In a meta-analysis of 19 RCTs in 1242 patients with DPN, the efficacy of oral ALA (600, 1200, and 1800 mg/day) was evaluated based on TSS, neurological disability score (NDS), neuropathy impaired score (NIS), NIS-LL, vibration perception threshold (VPT), nerve conduction study (NCS) results, and global satisfaction (28). ALA treatment produced favorable results for TSS (a dose-related trend was observed), NDS, and the global satisfaction score but not for VAS, VPT, NIS-LL, and NCS (28). Also, in a small cohort of 20 patients, ALA improved night pain, paresthesia, muscle atrophy and difficulty in walking (142).

In the NATHAN 1 (The Neurological Assessment of Thioctic Acid in Diabetic Neuropathy) trial, neuropathic deficits were improved after 4 years of ALA treatment in patients with mild to moderate largely asymptomatic DPN (143). In a *post-hoc* analysis of the NATHAN 1 trial, the use of ALA (vs. placebo) was associated with better outcome in terms of NIS-LL following 4-year treatment in patients with mild-to-moderate DPN, which was predicted by higher age, lower BMI, male sex, normal blood pressure, history of cardiovascular disease (CVD), insulin treatment, longer duration of diabetes and neuropathy, and higher neuropathy stage (144). Thus, ALA is considered effective in the amelioration of neuropathic impairments in elderly insulin-treated patients with history of CVD in whom weight and blood pressure are well controlled, as well as in those with more severe stages of diabetes and neuropathy, and HbA1c levels ≥7% (144). Although there is little evidence from studies, the participating experts consider that poor glycemic control leads to higher rate and a worse prognosis of macro- and micro-vascular complications. Without achieving the glycemic control, it is impossible to prevent complications and to slow progression in any patient. Therefore, it is not possible for any of the agents used in treatment of neuropathy to prevent complications as long as the glucolipotoxicity persists.

In a meta-analysis of 9 studies of DPN, compared to placebo, oral ALA treatment revealed a reduction in the NIS (muscle weakness, reflex loss, touch pressure, vibration, joint position, and motion), NIS-LL (motor nerve function and reflexes) and NDS (cranial nerve damage, muscle strength, reflex loss, and loss of sensation), besides the TSS (145). Accordingly, oral ALA treatment is considered a favorable option for DPN in terms of improving pain symptoms, and motor and nerve damage along with excellent safety profile (145–147). In a real-world cohort of 443 diabetic patients with chronic painful neuropathy who were treated with ALA 600 mg qd orally for a mean period of 5 years, long-term therapy with ALA acid was considered a safe and effective treatment option in outpatients with DPN (148). Notably, stopping ALA after 5 years of treatment yielded the recurrence of symptoms after 2 weeks, while switching from long-term treatment with ALA to central analgesic drugs such as gabapentin was associated with considerably higher rates of side effects, frequencies of outpatient visits, and daily costs of treatment (148). These findings were considered to indicate that treatment of DPN is a long term treatment, even at symptom-free intervals, which requires a drug with pathogenetic properties like ALA (148).

In addition, patients with diabetes were reported to have decreased levels of circulating ALA, supporting the beneficial effects of ALA supplementation in the management of DPN (132, 144).

Notably, in a recent meta-analysis of 24 RCTs in patients with metabolic diseases, ALA was also found to improve glucose homeostasis (decreased FBG, insulin, HOMA-IR and HbA1c) and lipid profile (decreased triglycerides, total cholesterol and LDL-cholesterol) (149). The potential mechanisms underlying its ability to improve glucose homeostasis have been demonstrated in some studies, mainly based on ALA-mediated increase in glucose uptake in peripheral tissues (i.e., muscles) in obese animals (150, 151) and stimulation of glucose uptake by translocation of glucose transporters to plasma membrane and increasing the tyrosine phosphorylation of insulin receptor substrate-1 (152).

Also, its effect on DPN is considered to be greater when used as part of a conventional treatment (i.e. gliclazide, SGLT2i, metformin, and GLP 1 analogs) in T2D patients experiencing neuropathic pain (153, 154) (**Box 2**).

Box 2 **Key points – ALA as a pathogenesis-directed antioxidant therapy for DPN (132–135, 138–147, 153, 154).**

- ALA is a first-line disease-modifying antioxidant therapy for DPN, given that it is a direct and indirect antioxidant that works with a strategy targeted directly against ROS and indirectly in favor of endogenous antioxidant capacity (glutathione) for improving DPN conditions.
- Its efficacy has been consistently demonstrated in many clinical trials, real-word studies and meta-analyses in the setting of DPN, in terms of improvement in motor and sensory nerve conduction velocity and amelioration of neuropathic symptoms (stabbing pain, burning pain, paresthesia, and asleep numbness of the feet).
- Specifically, ALA treatment was associated with a reduction in the NIS (muscle weakness, reflex loss, touch pressure, vibration, joint position, and motion), NIS-LL (motor nerve function and reflexes) and NDS (cranial nerve damage, muscle strength, reflex loss, and loss of sensation), besides the reduction in the TSS
- Long-term therapy with ALA is considered a safe and effective treatment option in patients with DPN.
- The recurrence of symptoms after 2 weeks of treatment discontinuation in patients who were on ALA therapy for 5 years, and the association of switching from long-term treatment with ALA to central analgesic drugs such as gabapentin with considerably higher rates of side effects, frequencies of outpatient visits, and daily costs of treatment, indicate that treatment of DPN is a long term treatment, even at symptom-free intervals, which requires a drug with pathogenetic properties like ALA.
- ALA seems to be helpful in improving glucose homeostasis (decreased FBG, insulin, HOMA-IR and HbA1c) and lipid profile (decreased triglycerides, total cholesterol and LDL-cholesterol), and its effect on DPN is considered to be greater when used with conventional treatment in T2D patients experiencing neuropathic pain.

### 6.3 Symptomatic treatment of painful DSPN

#### 6.3.1 Pharmacological treatment

For pharmacological treatment of painful DPN, serotonin-norepinephrine reuptake inhibitors (SNRIs: duloxetine and venlafaxine), tricyclic agents (TCAs: amitriptyline, imipramine) and gamma-aminobutyric acid (GABA) analogues (gabapentin or pregabalin) are considered as the first-line agents, while the combination therapy (antidepressant combined with gabapentin), topical treatments and opioids are recommended as the second-line or third-line agents (97, 128, 155–157). Recently, mirogabalin, a third member of gabapentinoids with distinct binding characteristics of voltage-gated calcium channels, was approved for use in DPN in Japan, Korea, Taiwan, and China (158).

The antioxidant ALA (IV form in particular) is also an effective treatment in amelioration of neuropathic pain, and even in reducing the use of rescue drugs (i.e., pregabalin, duloxetine, and tapedantol) (8, 52, 129, 135, 138, 139, 143–145, 159). Notably, in a retrospective analysis of the nationwide database in Hungary, a comparison of propensity matched cohorts of DPN patients treated with ALA vs. those treated with symptomatic analgesic pharmacotherapies revealed the lower occurrence of cardiovascular and cerebrovascular morbidity (acute myocardial infarction, stroke and hospitalization for heart failure), cancer events and all-cause mortality in patients treated with pathogenetically oriented ALA, while no significant change was observed in hazard for lower limb amputation (146). The duration of follow up treatment was also significantly longer with ALA than with symptomatic pharmacotherapies (146, 147).

When choosing a particular agent for treating painful DPN, certain factors besides the potential efficacy of the drug should be considered, such as existing comorbidities, potentials side effects of the medication, drug interactions and cost (8, 49, 97, 155, 160). The commonly prescribed drugs for painful DPN are summarized in **Table 3**, with regard to doses and comorbidity-based contraindications (8, 97, 155, 160).

**Table 3:** Pharmacological treatment of painful DPN (8, 95, 151, 156).

Pharmacological treatment of painful DPN	
**TCAs:** Amitriptyline (25-150 mg/day), Imipramine 25-150 mg/day	
**SNRIs:** Duloxetine (0-120 mg/day)^a^	
**GABA analogs:** Pregabalin (300-600 mg/day)^a^, Gabapentin (300-3600 mg/day)	
**Opiates:** Tramadol (200-400 mg/day), Oxycodone (20-80 mg/day), Morphine sulphate SR (20-80 mg/day), Tapentadol ER (100-400 mg/day)	
Alpha-lipoic acid^b^	
**Capsaicin cream (**0.075% applied sparingly 3-4 times per day)	
**Lidocaine (**5 mg/kg given intravenously over 1 hour with ECG monitoring)	
Tailored treatment	
*Factors*	*Contraindication*
Co-morbidities	
Glaucoma	TCAs
Orthostatic hypotension	TCAs
Cardiovascular disease	TCAs
Hepatic disease	Duloxetine
Edema	Pregabalin, gabapentin
Unsteadiness and falls	TCAs
Other factors	
Cost	Duloxetine, pregabalin
Weight gain	TCAs, pregabalin, gabapentin

GABA, gamma aminobutyric acid; SNRIs: Serotonin-norepinephrine reuptake inhibitors; TCAs, Tricyclic agents.

a Only duloxetine and pregabalin have been approved by both the European Medicines Agency and US Food and Drug Administration for the treatment of painful DPN.

b IV form is marketed only in Germany, Eastern Europe and China

However, despite these options, suboptimal pain relief is common with response to analgesic monotherapy achieved only by 50% of patients with painful DPN (8, 22, 98, 155, 161). Hence, insufficient response to a fist-line agent is not uncommon, necessitating a second first-line drug or a combination pharmacotherapy (gabapentinoids and antidepressants in particular) in those with treatment failure despite careful dose titration and adequate duration of first-line therapy (22, 49, 97, 154, 159).

#### 6.3.2 Non-pharmacological approaches

Because there is no entirely satisfactory pharmacotherapy of painful DSPN, non-pharmacological treatments such as psychological support, acupuncture, transcutaneous electrical nerve stimulation (TENS), percutaneous electrical nerve stimulation (PENS), low-intensity laser therapy and for severe resistant cases, electrical spinal cord stimulation and high-frequency repeated transcranial magnetic stimulation (rTMS) of the motor cortex can also be used, despite the relatively low level of evidence (4, 8, 22, 97, 162, 163).

Psychological interventions such as mindfulness-based stress reduction and cognitive behavioral therapy can also have a significant beneficial effect on pain severity, pain interference, depressive symptoms, and quality of life in patients with DPN (164).

#### 6.3.3 Vitamin supplements

Diabetic patients with chronic use of metformin and proton pump inhibitors (PPIs) are at increased risk of vitamin B12 deficiency, emphasizing the need for annual assessment of the vitamin B12 status in people with diabetes treated with metformin or PPIs (99, 165, 166).

The screening for vitamin B12 status in T2D patients with chronic metformin use is based on categories of low (<200 pg/mL), borderline (200–300 pg/mL) and normal (> 300 pg/mL) vitamin B12 levels, in accordance with normal values derived from one of the largest studies in the Diabetes Prevention Program Outcomes Study (48, 167).

Vitamin B12 supplementation in deficient patients with DPN was reported to be effective in reducing neurophysiological parameters, pain intensity and sudomotor function, and improving the quality of life (168–170). However, it should be noted that excessive vitamin B6 (pyridoxine) ingestion in the form of vitamin B1–6-12 combination tablets may also cause irreversible neurotoxicity (171).

While vitamin B12 deficiency is suggested to develop in a dose- and time-dependent manner in T2D patients on chronic metformin treatment (172), in a meta-analysis metformin use was not found to correlate with a high incidence of neuropathy (165). Likewise, in a longitudinal 9-year follow-up study assessing the interaction between vitamin B12 deficiency, DPN, and metformin exposure in diabetes, people with vitamin B12 deficiency (vs. those without deficiency) had an increased hazard of developing DPN, while people with DPN (vs. those without DPN) had increased hazard of developing vitamin B12 deficiency (173). Metformin significantly increased the risk of DPN in patients with vitamin B12 deficiency, whereas metformin treatment in preexisting DPN patients did not increase but rather reduced the risk of vitamin B12 deficiency (173).

Hence, further prospective studies addressing the incidence of DPN in relation to the degree of vitamin B12 deficiency per daily and cumulative doses of metformin in each therapeutic period are necessary to clarify the complex relationship between metformin treatment, vitamin B12 deficiency, and DPN occurrence in T2D (173) (**Box 3**).

Box 3 **Key points – Treatment options and future perspectives (3, 4, 18, 97, 127, 131–135, 173).**

- First-line interventions for DPN is currently represented by optimized glycemic control (mainly for T1D) and multifactorial intervention (mainly for T2D), along with lifestyle optimization and weight management.
- There is a need for personalized treatment/mechanism-based approaches for DPN, which includes the targeted interventions based on the comorbid risk factors and underlying disease mechanisms specific to each patient, besides the optimal diabetes treatment
- ALA is an effective first-line disease-modifying antioxidant therapy for long-term management of DPN.
- There is still a gap in existing research in the field, necessitating well-designed, robust, multicenter clinical trials, with sensitive endpoints and standardized protocols to facilitate the diagnosis of DPN via a simple and effective algorithm and to track progression of disease and treatment response.
- Identification of novel risk and prognostic biomarkers may enable an individualized treatment pathway for patients with DPN from a potentially disease-modifying perspective, and provide onward opportunities for novel treatments that would be efficacious in early stages of DPN, and alter the natural course of the disease.

## 7 Conclusions

The participating experts consider the suspicion of the disease by clinicians as the key factor, emphasizing the improved awareness of the disease during the patient journey by the first admission or referring physicians as well as the increased familiarity of non-specialist physicians with recognition of DPN. The proposed screening and diagnostic algorithm involves the consideration of clinical DPN in a patient with prediabetes or diabetes who presents with neuropathic symptoms and/or signs of neuropathy in the presence of DPN risk factors, with careful consideration of laboratory testing to rule out other causes of distal symmetric peripheral neuropathy and referral for a detailed neurological work-up for a confirmative test of either small or large nerve fiber dysfunction only in atypical cases. There is still a gap in existing research in the field, necessitating well-designed, robust, multicenter clinical trials with sensitive endpoints and standardized protocols to facilitate the diagnosis of DPN via a simple and effective algorithm and to track the progression of disease and treatment response. Although, the first-line interventions for DPN are currently represented by optimized glycemic control (mainly for T1D) and multifactorial intervention (mainly for T2D), along with lifestyle optimization and weight management, there is a need for individualized pathogenesis-directed approaches targeting specific underlying mechanisms involved in the disease. ALA seems to be an important first-line disease-modifying and pathogenesis-directed agent in this regard, given that it is a direct and indirect antioxidant that works with a strategy targeted directly against ROS and indirectly in favor of endogenous antioxidant capacity (glutathione) for improving DPN conditions. Identification of biomarkers/predictors that would allow an individualized approach from a potentially disease-modifying perspective may provide onward opportunities for novel treatments that would be efficacious in early stages of DPN and alter the natural course of the disease.

In this regard, this expert opinion document is expected to increase awareness among physicians about conceptual, clinical, and therapeutic aspects of DPN and to assist them in timely recognition of DPN and translating this information into their clinical practice for best practice in the management of patients with DPN.

## Author contributions

AA: Conceptualization, Investigation, Methodology, Project administration, Writing – original draft, Writing – review & editing. AK: Conceptualization, Investigation, Methodology, Project administration, Writing – original draft, Writing – review & editing. IS: Conceptualization, Investigation, Methodology, Project administration, Writing – original draft, Writing – review & editing. ISS: Conceptualization, Investigation, Methodology, Project administration, Writing – original draft, Writing – review & editing. RO: Conceptualization, Investigation, Methodology, Project administration, Writing – original draft, Writing – review & editing. HE: Conceptualization, Investigation, Methodology, Project administration, Writing – original draft, Writing – review & editing. MA: Conceptualization, Investigation, Methodology, Project administration, Writing – original draft, Writing – review & editing. HS: Conceptualization, Investigation, Methodology, Project administration, Writing – original draft, Writing – review & editing. TD: Conceptualization, Investigation, Methodology, Project administration, Writing – original draft, Writing – review & editing.
